# Chest pain with ST segment elevation in a patient with prosthetic aortic valve infective endocarditis: a case report

**DOI:** 10.1186/1752-1947-5-408

**Published:** 2011-08-24

**Authors:** Vishal Luther, Refai Showkathali, Reto Gamma

**Affiliations:** 1 Department of Medicine, Whittington Hospital NHS Trust, Magdala Avenue, London, N19 5NF, UK; 2 Department of Cardiology, The Essex Cardiothoracic Centre, Nethermayne, Basildon, Essex, UK, SS16 5NL, UK

## Abstract

**Introduction:**

Acute ST-segment elevation myocardial infarction secondary to atherosclerotic plaque rupture is a common medical emergency. This condition is effectively managed with percutaneous coronary intervention or thrombolysis. We report a rare case of acute myocardial infarction secondary to coronary embolisation of valvular vegetation in a patient with infective endocarditis, and we highlight how the management of this phenomenon may not be the same.

**Case presentation:**

A 73-year-old British Caucasian man with previous tissue aortic valve replacement was diagnosed with and treated for infective endocarditis of his native mitral valve. His condition deteriorated in hospital and repeat echocardiography revealed migration of vegetation to his aortic valve. Whilst waiting for surgery, our patient developed severe central crushing chest pain with associated anterior ST segment elevation on his electrocardiogram. Our patient had no history or risk factors for ischaemic heart disease. It was likely that coronary embolisation of part of the vegetation had occurred. Thrombolysis or percutaneous coronary intervention treatments were not performed in this setting and a plan was made for urgent surgical intervention. However, our patient deteriorated rapidly and unfortunately died.

**Conclusion:**

Clinicians need to be aware that atherosclerotic plaque rupture is not the only cause of acute myocardial infarction. In the case of septic vegetation embolisation, case report evidence reveals that adopting the current strategies used in the treatment of myocardial infarction can be dangerous. Thrombolysis risks intra-cerebral hemorrhage from mycotic aneurysm rupture. Percutaneous coronary intervention risks coronary mycotic aneurysm formation, stent infections as well as distal septic embolisation. As yet, there remains no defined treatment modality and we feel all cases should be referred to specialist cardiac centers to consider how best to proceed.

## Introduction

Atherosclerotic plaque rupture within a coronary vessel can lead to rapid vessel occlusion and subsequent myocardial ischaemia and necrosis [1]. Risk factors for the development of atherosclerosis include hypertension, diabetes mellitus, high cholesterol, a history of smoking, and a family history of atherosclerotic disease [2]. Current treatment involves either percutaneous coronary intervention (PCI) to relieve the occlusion, or thrombolysis to dissolve the occlusion [3].

There are more rare causes of acute myocardial infarction (AMI). We present and discuss the case of a patient with AMI secondary to embolisation of vegetation sitting on a prosthetic aortic valve in a patient with confirmed aortic valve infective endocarditis (IE).

## Case presentation

**Figure 1:**
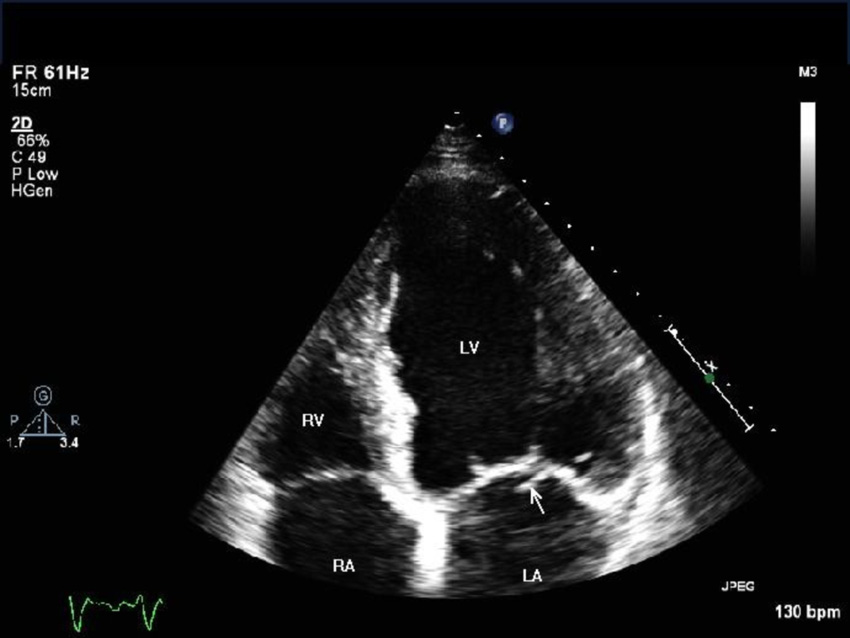
**Echocardiogram (apical view) showing vegetation in the native posterior mitral valve leaflet (white arrow)**. LA = left atrium; LV = left ventricle; RA = right atrium; RV = right ventricle.

**Figure 2:**
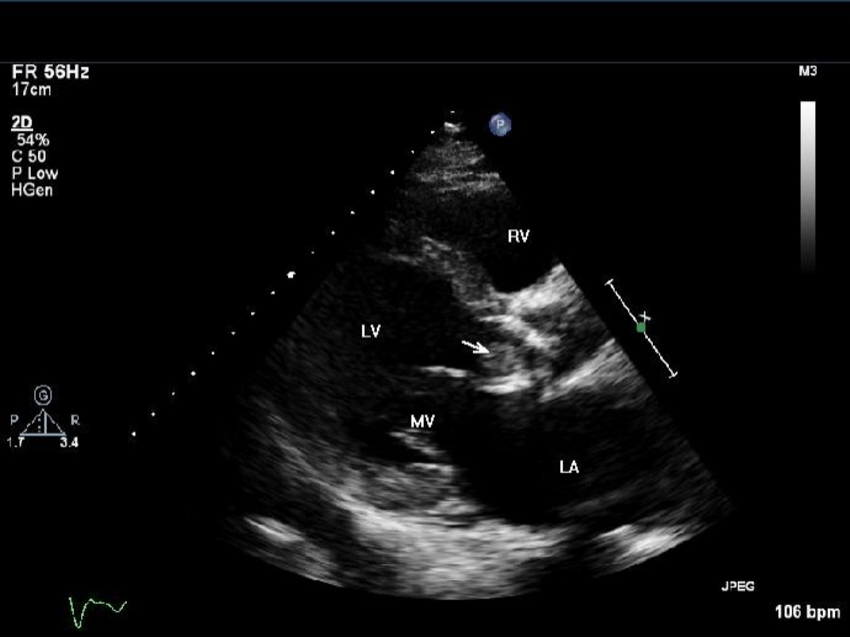
**Echocardiogram (parasternal long axis view) showing large vegetation in the tissue prosthetic aortic valve (white arrow)**. LA = left atrium; LV = left ventricle; MV = mitral valve; RV = right ventricle.

A 73-year-old British Caucasian man who had undergone a tissue aortic valve replacement five years previously was admitted to his local hospital with a two-week history of breathlessness, general malaise and night sweats. On examination, he was found to have an ejection systolic murmur in the aortic area and a pan-systolic murmur in the mitral area radiating to the axilla. His white cell count was elevated (15.1 × 10^9 ^cells/L, neutrophils 10.7 × 10^9 ^cells/L) and he had a raised C-reactive protein level of 101 mg/dL. The results of three consecutive blood cultures samples were negative even after five days in the culture media. His trans-thoracic and trans-oesophageal echocardiogram (ECG) results demonstrated vegetation involving the native posterior mitral valve leaflet (Figure 1) with moderate mitral regurgitation and a moderately stenosed tissue aortic valve. Vancomycin, Gentamicin and Rifampicin were given under microbiology guidance. Five days later, our patient became more unwell, and was found to be in worsening cardiac failure. A repeat echocardiogram showed the known vegetation on the mitral valve and new vegetation on the aortic valve of 1.5 cm (Figure 2) causing moderate aortic regurgitation. Our patient was subsequently transferred to our center for valve surgery.

**Figure 3:**
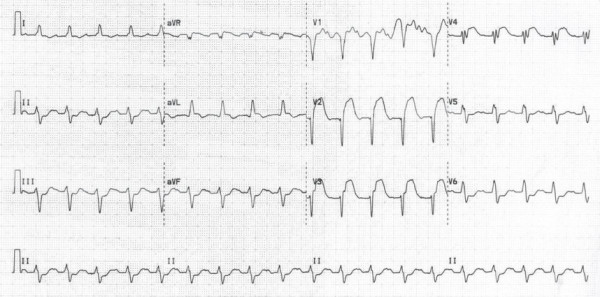
**Electrocardiogram showing ST elevation in V1 to V4 leads**.

Whilst awaiting surgery, our patient developed severe central crushing chest pain with associated anterior segment ST elevation on his ECG (Figure 3). Our patient had no previous history of angina, and was a non-smoker with no other cardiac risk factors. A coronary angiogram performed five years ago prior to his valve surgery revealed unobstructed coronaries. The most likely explanation for this ST segment elevation myocardial infarction (STEMI) was coronary embolisation of either part of the vegetation or thrombus attached to the vegetation. Thrombolysis is relatively contraindicated in this scenario. PCI risked mycotic aneurysm formation and either further systemic or coronary embolisation. Therefore, urgent surgical intervention was planned; however, our patient deteriorated rapidly and unfortunately died.

## Discussion

Coronary embolisation is a rare cause of AMI and needs to be considered in patients with atrial fibrillation, prosthetic heart valves, dilated cardiomyopathy, and IE, where either thrombus or vegetation can embolize into the coronary circulation. Although systemic embolisation can occur in up to 50% of cases of IE [4], coronary embolisation rate is about 0.3% [5]. There appears to be an increased risk of embolisation with vegetations that are > 1 cm in diameter, as in our patient's case [6]. Successful strategies that have been used to manage coronary embolisation in non-endocarditic patients include thrombolytics [7], PCI and thrombus aspiration [8].

There is no clear evidence available about the best treatment option for patients with coronary embolisation in the setting of acute IE [9]. Thrombolytic treatment of septic coronary embolisation is associated with an increased risk of cerebral vascular hemorrhage due to bleeding from silent cerebral microinfarctions or mycotic aneurysms [10]. Indeed AMI caused by septic embolisation is a relative contraindication to the use of thrombolytic agents. PCI involves coronary balloon angioplasty and stent deployment, and this risks mycotic aneurysm formation at the dilatation site. This occurs as the balloon crushes vegetation against the vessel wall [11]. Implanting foreign stent material into an infective site can lead to stent infection, and this can require stent excision and debridement [12]. In addition, PCI risks further distal vegetation embolisation [13]. As reported in a previous case report, 'the impulse to follow conventional strategies for coronary reperfusion should be tempered by thoughts of possible consequences' [11].

Surgical intervention in left-sided IE is in fact recommended in the context of systemic embolisation [14]. However, evidence of successful surgical intervention in the context of coronary embolisation is scarce, with a few case reports demonstrating success through coronary embolectomy [15].

## Conclusions

This case report presents a common condition seen in an uncommon setting. AMI is common, and the management is well defined and performed by acute physicians and cardiologists. However, in the absence of risk factors for ischaemic heart disease, clinicians need to consider alternate causes of AMI.

This is especially important in the case of septic coronary embolisation in patients with IE, as adopting the current strategies used in the management of myocardial infarction can be dangerous. Where suspicion is high, care should be urgently transferred to specialist cardiac centers where both interventional and surgical skills are available to decide on how best to proceed.

## Consent

Written informed consent was obtained from the patient's next-of-kin for publication of this case report and any accompanying images. A copy of the written consent is available for review by the Editor-in-Chief of this journal.

## Competing interests

The authors declare that they have no competing interests.

## Authors' contributions

VL wrote the initial draft of the case report. RS edited the case report and selected all the images to use. RG was our patient's consultant. All authors read and approved the final manuscript.
